# Public health round-up

**DOI:** 10.2471/BLT.19.010819

**Published:** 2019-08-01

**Children caught up in Pakistan’s HIV outbreak**

A mother with her child in Larkana district, Sindh province, Pakistan. HIV screening began in April at the main hospital in Ratodero Taluka in Larkana district and was expanded to other health facilities. By 28 June 2019, 876 people had tested HIV positive, 82% of whom are children under the age of 15.

**Figure:**
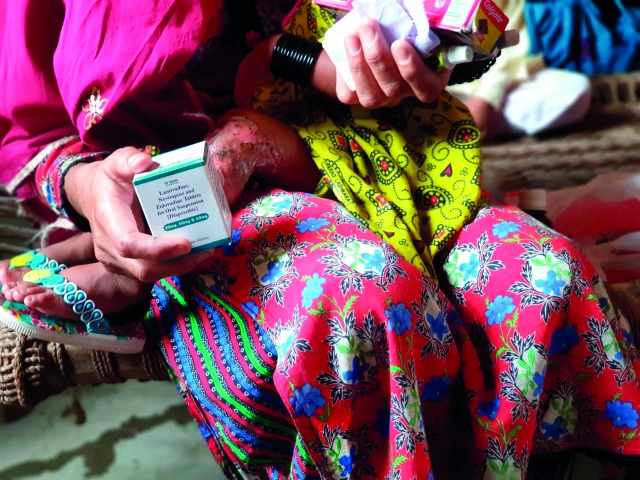
UNAIDS

## Pakistan HIV outbreak

The World Health Organization (WHO) mapped high-risk areas and health-system failures in the diagnosis, care and treatment of human immunodeficiency virus (HIV) infections in Larkana district, Sindh province, Pakistan. Multiple risk factors were identified that include: unsafe intravenous injections during medical procedures; unsafe child delivery practices; unsafe practices at blood banks; poorly implemented infection control programs; and improper collection, storage, segregation and disposal of hospital waste.

The outbreak investigation was done in response to reports of children newly diagnosed with HIV in April in Larkana district.

HIV screening began in April at the main hospital in Ratodero Taluka in Larkana district and was subsequently expanded to other health facilities including selected rural health centers and basic health units.

By 28 June, it was reported that 30 192 people had been tested. In total, 876 people tested positive, including 719 children under 15 years of age.

This is the fourth reported HIV outbreak in Larkana district since 2003. The first affected people who inject drugs, the second and third, both in 2016, mostly affected hospitalized patients.

The public health response being implemented by the provincial Department of Health and the Sindh AIDS Control Program, with the support of partners, includes the closing of unauthorized laboratories, blood banks and clinics, and the opening of a new antiretroviral therapy center for children at the Shaikh Zaid Hospital in Larkana district.

https://www.who.int/csr/don/03-july-2019-hiv-cases-pakistan/en/

## New essential medicines and diagnostics lists 

WHO published a new edition of its *Model list of essential medicines* and its *2^nd^ Model list of essential in vitro diagnostics* 9 July.

The lists are WHO’s core guidance documents to help countries prioritize health products. Inclusion on these lists mean that the products should be made available and affordable in each country’s health system.

Twenty-eight medicines for adults and 23 for children were added to the medicines list. The list also specifies new uses for 26 products, bringing the total to 460 products deemed essential for addressing public health needs.

Twelve new cancer therapies were added, including two recently developed immunotherapies (nivolumab and pembrolizumab), which have resulted in improved outcomes for people with advanced melanoma.

“The inclusion in this list of some of the newest and most advanced cancer drugs is a strong statement that everyone deserves access to these life-saving medicines, not just those who can afford them,” said WHO Director-General Tedros Adhanom Ghebreyesus.

Three new antibiotics were added for resistant infections and the AWaRe classification was updated. The AWaRe tool was developed to support efforts to contain rising resistance and ensure safe and effective use of antibiotics. The classification divides antibiotics into three categories: "Access," "Watch," and "Reserve" and provides indications for use in each category.

Twelve tests designed to detect a wide range of solid tumors were added to the *2^nd^ Model list of essential in vitro diagnostics.*

https://www.who.int/news-room/detail/09-07-2019-who-updates-global-guidance-on-medicines-and-diagnostic-tests-to-address-health-challenges-prioritize-highly-effective-therapeutics-and-improve-affordable-access

## Ebola reaches Goma

The first case of Ebola in the current outbreak was confirmed on 14 July in Goma, a city of about 1 million people in the Democratic Republic of the Congo. 

While the outbreak was still mainly confined to the provinces of North Kivu and Ituri, WHO assessed the risk of spread to neighbouring provinces and countries as very high. 

As of 15 July, about 3000 health workers had been vaccinated against the disease in Goma.

The United Nations hosted a high-level meeting in Geneva to review the situation on July 15. 

The meeting was chaired by the WHO Director-General Tedros Adhanom Ghebreyesus and UN Under-Secretary-General for Humanitarian Affairs and Emergency Relief Coordinator Mark Lowcock. The Democratic Republic of the Congo’s Minister of Health, Oly Ilunga and Minister for Solidarity and Humanitarian Action, Bernard Biando Sango, also attended.

“Together with the government, we can and will end this outbreak. We have better public health tools than ever to respond to Ebola, including an effective vaccine,” said Dr Tedros. “But we need to see an end to the attacks and other disruptions to the response.” 

Since January, there have been 198 attacks against the health response that have resulted in 7 deaths and left 58 health-care workers and patients injured. 

https://www.who.int/news-room/detail/15-07-2019-high-level-meeting-on-the-ebola-outbreak-in-the-democratic-republic-of-the-congo-affirms-support-for-government-led-response-and-un-system-wide-approach

## Cholera vaccination campaign completed

A large-scale, two-dose cholera vaccination campaign was completed 8 July 2019 in 15 health districts in the four central provinces of the Democratic Republic of the Congo. The first dose had been delivered at the beginning of June. Targeting 1.2 million people over 1 year of age, the 5-day, door-to-door campaign was implemented by 2 632 vaccinators recruited mainly from local communities.

“This cholera vaccination campaign marks the intensification of our response in the Democratic Republic of the Congo ,” said Dr Matshidiso Moeti, WHO Regional Director for Africa, “WHO and our partners are working with national authorities to rollout the vaccine, which comes in addition to multiple interventions introduced since the beginning of the cholera epidemic, including sanitation and water quality control in the affected areas, many of which have little access to a safe water supply.”

In 2018 the Democratic Republic of the Congo reported a cumulative total of 29 304 suspected cholera cases and more than 930 deaths. In 2019, 12 247 suspected cases of cholera and 279 deaths have been reported.

https://afro.who.int/news/more-million-people-be-vaccinated-phase-2-huge-cholera-vaccination-campaign-democratic

**Cover photo**

People from Venezuela cross the Tachira River which forms the border with Colombia. Around 2.7 million people have left the country since 2015, fleeing hyperinflation, food and power shortages, political turmoil, violence and persecution.

**Figure:**
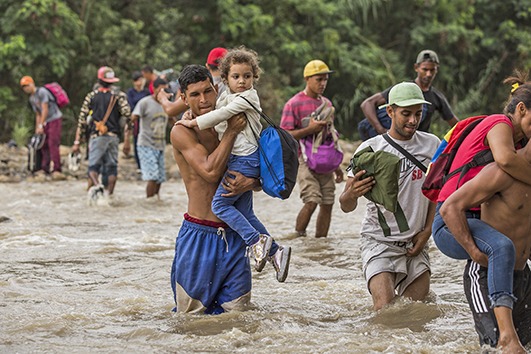
UNHCR/Vincent Tremeau

## Vaccine-derived polio in Angola

A new case of genetically-distinct circulating vaccine-derived poliovirus type 2 (cVDPV2) has been confirmed in Cuvango district, Huila Province, central Angola. This is the second cVDPV2 outbreak in Angola in 2019, occurring over 1 000 kilometers away from the first event.

The Global Polio Laboratory Network notified WHO of the case on 3 July 2019.

The first case was reported in early June in Lunda Norte Province, bordering the Democratic Republic of the Congo where two genetically-distinct outbreaks of cVDPV2 were previously reported.

The identification of cases in Angola and the Democratic Republic of the Congo highlights the risk of cVDPV2 spreading across borders, especially given the high population mobility, inadequate sanitation and low immunization coverage in the countries concerned.

WHO and partners in the Global Polio Eradication Initiative are supporting the Ministry of Health of Angola with epidemiological and virologic investigations.

https://apps.who.int/iris/bitstream/handle/10665/325777/OEW27-0107072019.pdf

## Steep drop in trachoma estimates

The number of people at risk of trachoma – the world’s leading infectious cause of blindness – fell from 1.5 billion in 2002 to just over 142 million in 2019, a reduction of 91%.

The reduction is due to a combination of control measures in endemic countries, and more accurate estimates of people at risk.

The data were presented by WHO on 27 June at a meeting of the WHO Alliance for the Global Elimination of Trachoma by 2020.

WHO also reported that the number of people requiring surgery for trachomatous trichiasis – the late, blinding stage of trachoma – has dropped from 7.6 million in 2002 to 2.5 million in 2019, a reduction of 68%.

Trachoma remains endemic in 44 countries and is estimated to have rendered 1.9 million people blind or visually impaired.

https://www.who.int/news-room/detail/27-06-2019-eliminating-trachoma-who-announces-sustained-progress-with-hundreds-of-millions-of-people-no-longer-at-risk-of-infection

**Looking ahead**

12 September - Global Vaccination Summit. Brussels, Belgium.

23 September - High-Level Meeting on Universal Health Coverage. New York, United States of America.

24–25 September - Sustainable Development Goals Summit, New York, United States of America.

